# The Book World of Medicine and Science

**Published:** 1905-06-10

**Authors:** 


### The Book World of Medicine and Science.

The History of the Society of Apothecaries of London. By C. R. B. Barrett, M.A. Illustrated by the Author. (London: Elliot Stock. 1905. Crown 4to. Pp. xxxix. and 310. Price £1 1s. net.)

The whole duty of an apothecary was summed up by Doctor Bullein in the reign of Queen Elizabeth in the following words:—" The Apothecary must first serve God, be cleanly and pity the poor. He must not be suborned for money to hurt mankind. His place of dwelling and shop are to be cleanly to please the senses withal. His garden must be at hand with plenty of herbs, seeds and roots to sow, plant, gather, preserve and keep them in due time. He must choose medicines by colour, taste, odour and figure. He must have his mortars, stills, pots, filters, glasses and boxes clean and sweet. He must have his charcoals at hand to make decoctions, syrups, etc. He should have two places in his shop, the one most clean for the Physic and a baser place for Chirurgery stuff. He must neither increase nor diminish the physician's bill (prescription) and must keep it for his own discharge. He must neither buy nor sell rotten drugs. He must peruse often his wares that they corrupt not. He must not put in *quid pro quo* without advisement. He may open well a vein for to help the pleurisy but he must remember that his office is only to be the Physician's cook. He must use true weight and measure. He must remember his end and thus 'I do commend him to God if he be not covetous or crafty, seeking his own lucre before other men's health, succour and comfort.' " These delightful admonitions give us in a few words the points upon which the apothecaries prided themselves at a time when as yet they were bound to the grocers in a somewhat unholy alliance. This was severed in 1617 and King James I., when the grocers petitioned against the separation of the apothecaries from their body, replied to them in the following scathing words:—" Another grievance of mine is that you have condemned the patents of the apothecaries in London. I myself did devise that corporation and do allow it. The grocers who complain of it are but merchants. The mystery of these apothecaries were belonging to the apothecaries, wherein the grocers are unskilful, and therefore I think it fitting they should be a corporation of themselves. The merchants bring home rotten wares from the Indies, Persia, and Greece, and here with their mixtures make waters, and sell such as belong to the apothecaries, and think no man must control them because they are not apothecaries." It is clear, therefore, that at the end of the sixteenth and beginning of the seventeenth centuries the apothecaries of the better class made a determined and successful attempt to raise themselves in the social scale and from tradesmen to become a profession.

The countenance shown to the Society of Apothecaries by James I. aided them greatly in their early struggles, and the interesting history written by Mr. Barrett tells the story in detail by extracts from the very complete set of minute books which have been kept by the Society. The tale is that which is usually told of any successful body. A small and resolute band of men carry a majority of their lukewarm colleagues along with them by sheer force of character until they attain the end they have had in view. Poor at first they amass money in the course of a few generations, and are then enabled to launch out into larger speculations. The Society of Apothecaries have always been fortunate in possessing men of sufficiently wide views to enable the Society to take advantage of every opportunity as it offered. It thus happens that nearly 300 years after its foundation the Society still fulfils the hopes of its founders. It has a good garden for herbs, it sells pure drugs, it remembers that it is the physician's cook, it teaches its licentiates how to open well a vein for to help the pleurisy; in other words, it is one of the great licensing bodies in the medical profession, but though it still has a most clean place for physic, it no longer has a baser place for chirurgery stuff.

Mr. Barrett has illustrated his history with sketches in black and white. The cuts include the architectural features, the contents of the hall, many of the objects of interest in the building, and views of various picturesque nooks and corners of a part which is but little known to the large body of Londoners, even though they pass the very doors of the Apothecaries' Society every day of their lives. These illustrations add greatly to the value and interest of the book.

Lectures on Clinical Surgery. By H. C. Hindes, M.B., M.Ch. (London: Baillière, Tindall and Cox. 1905.)

These lectures, which appear to be the outcome of much careful and accurate clinical observation, are mainly devoted to the diagnosis and treatment of genito-urinary cases. The chapters on the use of the cystoscope are especially valuable and worthy of study. The author rightly appreciates the value of cystoscopy as a means of diagnosis in diseases of the urinary tract. Cystoscopy is one of the most difficult of clinical methods at our disposal, and for this reason the results obtained from it are often disappointing. The beautiful coloured illustrations of cystoscopic appearances which Mr. Hindes gives in this work should serve to encourage the more frequent employment of this instrument. In the article on the treatment of hypertrophied prostate it is interesting to note that Bottini's method of cutting a channel through the prostate by means of a cautery is described and advocated. The book is well produced, and the coloured plates are extremely good.

Poisonous Plants of All Countries. By Bernhard Smith. (Bristol: John Wright and Co. 1905. Price 2s. 6d.)

This is, in reality, a compendium and will be of use, as a book of reference, to pharmacologists and medical jurists. The two plates illustrating fungi are not well coloured.

Lateral Curvature of the Spine. By Richard Barwell, F.R.C.S. (London: Baillière, Tindall and Cox. 1905. Price 3s. net.)

Though the sixth edition of the work, this book has been almost entirely rewritten. By a special device the author has obtained full-length photographs of his patients, from which he seeks to demonstrate that the majority of cases of scoliosis are caused by pelvic malposture. This may be due to inequality in the length of the limbs produced by irregularity of growth or by joint disease, or it may be consequent to habitual pelvic obliquity produced by wrong attitudes assumed at rest. Another common cause for lateral curvature he considers to be a condition which he calls pelvic amesiality, in which the lower limbs do not stand vertically to the floor, but slope obliquely to the right or left.

